# Design and Research of an Articulated Tracked Firefighting Robot

**DOI:** 10.3390/s22145086

**Published:** 2022-07-06

**Authors:** Jianwei Zhao, Zhiwei Zhang, Shengyi Liu, Yuanhao Tao, Yushuo Liu

**Affiliations:** 1 School of Mechanical Electronic and Information Engineering, China University of Mining and Technology (Beijing), Beijing 100083, China; zhaojianwei@cumtb.edu.cn (J.Z.); sqt2000402039@student.cumtb.edu.cn (S.L.); sqt2100401017@student.cumtb.edu.cn (Y.T.); sqt2100402037@student.cumtb.edu.cn (Y.L.); 2 Institute of Artificial Intelligence, University of Science and Technology Beijing, Beijing 100083, China

**Keywords:** firefighting robot, four tracks and four drives, articulated tracked structure, ADAMS simulation

## Abstract

Aiming to improve the situation where a firefighting robot is affected by conditions of space and complex terrain, a small four-track, four-drive articulated tracked fire-extinguishing robot is designed, which can flexibly perform fire detection and fire extinguishing tasks in a narrow space and complex terrain environment. Firstly, the overall structure of the robot is established. Secondly, the mathematical model of the robot’s motion is analyzed. On this basis, the kinematics simulation is carried out by using ADAMS, and the motion of the robot is analyzed when it overcomes obstacles. Finally, the prototype was produced and tested experimentally. The robot has good obstacle-surmounting ability and excellent stability, is a reasonable size, and can perform various firefighting tasks well.

## 1. Introduction

With the continuous advancement of science and technology in today’s world, the types and incidences of safety accidents in various smelting, chemical, and aerospace industries are increasing [1,2,3]. The leakage, burning, and even explosions of various hazardous chemicals, flammable and toxic chemical substances, and radioactive substances occur from time to time, causing fire accidents to increase year by year [4]. The internal situation of such fires is also extremely complex. After the occurrence of a dangerous fire, the post-disaster terrain environment is complex and the space is small, which is not conducive to the entry of rescuers. At the same time, certain dangerous substances may remain in the environment, such as dust, chemicals, toxic fumes, toxic and harmful gases, etc., which are prone to secondary accidents [5,6,7]. Therefore, when entering an accident scene where there are great dangers such as flammable, explosive, and toxic gases to carry out rescue activities, firefighting robots can be used to replace firefighters and can efficiently complete a series of investigations and inspections, take care of smoke exhaust and cooling, and eliminate dangerous situations. It can more safely and efficiently complete the tasks of investigation and fire extinguishing, improving the personal safety and rescue ability of firefighters, which is of great significance to improve rescue efficiency [8,9,10,11,12].

To sum up, firefighting robots carry out investigations and firefighting tasks quickly, smoothly, and safely, improve the work efficiency of fire rescue personnel, and reduce casualties. It is necessary to develop robots with a superior obstacle jumping ability, reasonable scale, and superior performance that can replace or partially replace fire and rescue personnel in time. It is of great significance for the life safety of fire rescue personnel that the closed-space detection and firefighting robot enters the accident scene to carry out environmental investigations and firefighting tasks.

The United States and Japan are the first countries in the world to study and apply firefighting robots. After years of development, firefighting robots in the two countries have entered the era of intelligent control. In terms of control technology, the research stage of firefighting robots can be divided into three stages. In the first stage, firefighting robots only have basic remote control functions and firefighting functions to replace firefighting personnel in firefighting operations. The second stage adds the computer-aided function of environmental perception on the basis of the first stage, so that the robots have a certain exploration ability. The third stage is the intelligent stage of firefighting robots that have the ability of strong, autonomous perception, and which can make self-decisions and make predictions such as route avoidance according to the fire scene environment. At present, the firefighting robots in the third stage are high-level technical equipment and are still in their infancy.

**Figure 1:**
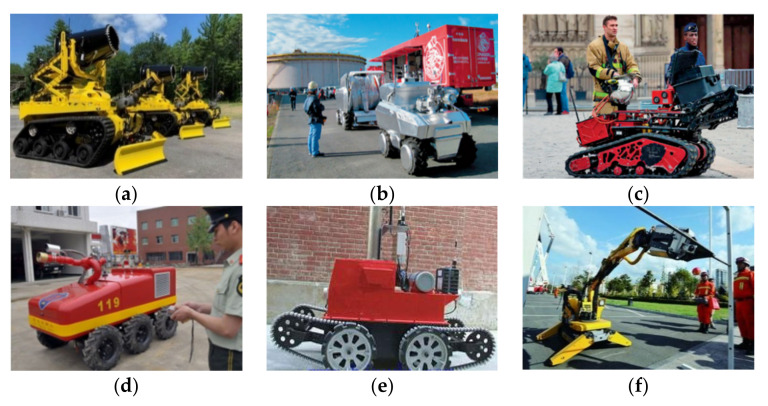
Firefighting robots in various countries. (**a**) Thermite RS3. (**b**) Scrum Force. (**c**) Giant Robot. (**d**) JMX.LT50/LD50. (**e**) JZX-GL/A. (**f**) Brokk-50.

Maine-based robot company Howe & Howe Technologies produced the Textron firefighting robot Thermite RS3, as shown in Figure 1a, which was acquired by the Los Angeles Fire Department in 2020. The robot moves using a traditional fixed-wheel tracking structure, which is bulky and weighs about 3500 lbs. According to Japanese media reports, the fire brigade of Japan’s first firefighting robot system “Scrum Force” was deployed to the fire station in Ichihara City, Chiba Prefecture. As shown in Figure 1b, the ground robot in this system is a wheeled robot, which is flexible and fast, but has a weak ability to cross obstacles. The French giant firefighting robot, as shown in Figure 1c, is a multi-functional, crawler-type, remote-controlled firefighting robot, which adopts a traditional fixed-crawler structure. The giant robot emerged in April 2019 in the firefighting and rescue operation of Notre Dame Cathedral in Paris. At present, the firefighting special robots that are widely used in China’s firefighting industry mainly include the following types: first, the JMX.LT50/LD50 firefighting robot as shown in Figure 1d, which is the first firefighting robot independently developed by China and which moves in a wheeled manner; the second is the JZX-GL/A firefighting reconnaissance robot as shown in Figure 1e, which has strong reconnaissance capabilities and adopts a joint crawler structure that gives it a strong ability to overcome obstacles; the third is the Brokk-50 firefighting demolition robot as shown in Figure 1f, which was based on and improved on a similar robot in Sweden, and also adopts the traditional fixed-crawler structure.

According to the task requirements of the firefighting machine, the firefighting robot needs to have a fast passing ability, excellent obstacle-crossing ability, and multiple detection ability. Considering the complexity of the terrain at the accident site, a four-track, four-drive articulated mobile mechanism is designed. The four-track, four-drive articulated mobile mechanism has stronger performance of obstacle crossing than the wheel moving mechanism and stronger stability than the traditional crawler moving mechanism. This mechanism enables the robot to have excellent obstacle-surmounting ability and stability when overcoming obstacles, and can better support the robot to complete firefighting tasks. Additionally, it is equipped with an undulating device, which can be freely retracted and undulated to provide a more convenient detection angle for the detection device.

The remainder of this paper is organized as follows. Section 2 describes the system architecture and design of the firefighting robot. Section 3 describes the mathematical model of the robot. Section 4 describes the analysis of the robot’s climbing ability and the analysis of the obstacle-climbing posture. Section 5 describes the ADAMS simulation analysis of the robot mobile chassis. Section 6 is the prototype experiment of the robot function, and Section 7 summarizes the work completed in this paper.

## 2. System Architecture and Design of Firefighting Robots

**Figure 2:**
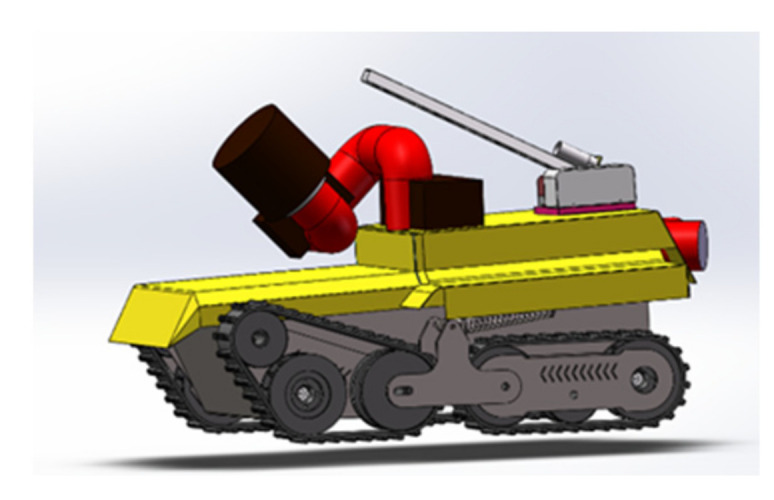
Overall appearance of the robot.

The main hardware structure design of the firefighting robot is mainly composed of three parts, namely, the chassis walking mechanism, the firefighting cannon device, and the undulating device. As shown in Figure 2. The walking mechanism with a reasonable size, flexible movement, and strong ability to surmount obstacles is an important element for the successful completion of dangerous accident detection and rescue tasks. The firefighting cannon mechanism is the main mechanism for the firefighting robot to perform fire extinguishing tasks. Through the fire cannon, the robot can replace the firefighters to spray firefighting agents. Sensors on the undulating device allow the robot to provide more flexible viewing angles when investigating accident conditions.

### 2.1. Robot Mobile Chassis

When a firefighting robot performs firefighting tasks, the most critical premise is that the robot can move flexibly in a complex terrain environment; thus, the design of the mobile system is an important element for the robot to complete various tasks. At present, the mobile carriers of firefighting robots include wheeled, crawler, footed, and bionic structures. A wheeled robot has the advantages of fast speed, strong flexibility, and easy control. However, the wheeled load capacity is small and the obstacle-crossing ability is poor. The multi-legged robot can walk or jump in a stable manner in uneven areas, and can climb over obstacles or overcome large obstacles. Although it is highly adaptable to the environment, its manufacture and control are complicated and its movements are slow. Bionic robots move in the same way as living creatures in nature, such as snake-like and humanoid robots. The former can move freely and flexibly like a snake, whereas the latter can more flexibly replace humans in operations and have a more flexible way of moving. However, the effective bearing capacity is not strong, and the structure is complex. Although the moving speed of the crawler robots is slower than that of the wheeled robots, the speed is generally in between the wheeled and the footed robots. The control of crawler robots is simple and the ability to surmount obstacles is strong [13,14,15,16]. To summarize, the crawler robots have significant advantages in dealing with accidents and dangers, and this structure is the most suitable for this design.

**Figure 3:**
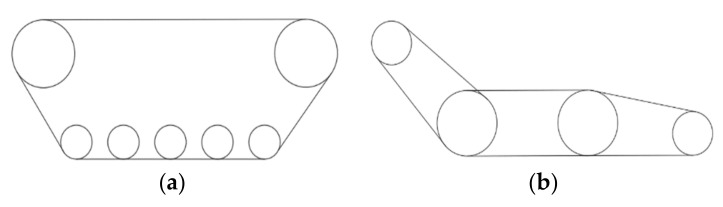
Traditional track structure. (**a**) Fixed-wheel track mechanism. (**b**) Articulated track structure.

**Figure 4:**
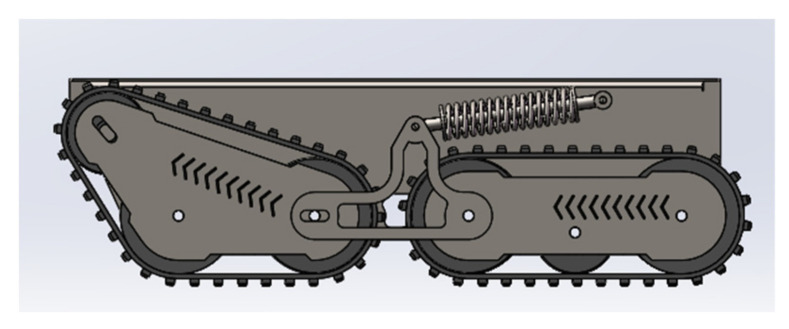
Robot chassis structure diagram.

The traditional crawler structure is divided into a fixed type and articulated type, as shown in Figure 3. The moving speed of the fixed crawler is faster than that of the articulated type, but the ability of the articulated type to overcome obstacles is relatively strong. Considering the complex terrain and environmental factors, the latter robot uses a four-track, four-drive joint structure, with spring shock absorbers added at the joints, as shown in Figure 4, to allow the robot to have a strong obstacle-surmounting ability and strong stability. Furthermore, this chassis can provide a certain horizontal angle for the top platform when overcoming obstacles.

### 2.2. Firefighting Cannon

**Figure 5:**
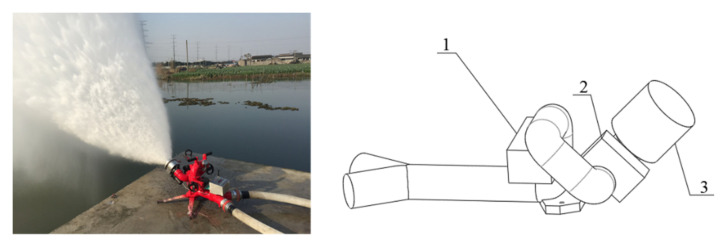
Firefighting cannon structure diagram.

The firefighting cannon can carry out continuous large-flow, long-range operations at the fire scene, and can play an irreplaceable role in actual combat. This design used the PSKDY40ZB mobile self-swing electronically controlled firefighting cannon, which has the characteristics of being long range, and having a remotely operated cannon body and cannon head. The main structure of the firefighting cannon is controlled by three motors, as shown in Figure 5. The serial numbers 1, 2, and 3 in Figure 5 represent the three drive motors. The No. 1 motor is responsible for the left and right rotation of the cannon, the No. 2 motor controls the elevation angle of the cannon, and the No. 3 motor controls the degree of atomization of the firefighting agent. The three motors of the firefighting cannon are controlled by remote control, and the control distance can reach 3.5 km.

### 2.3. Undulating Device

**Figure 6:**
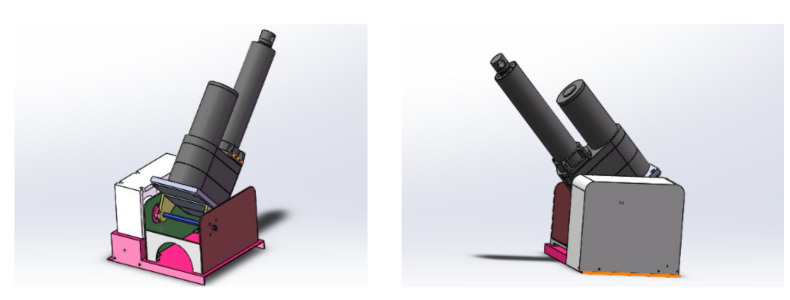
Undulating device.

The undulating device is mainly composed of the bottom seat of the undulating device and an electric push rod, as shown in Figure 6. A worm gear reducer and a stepping motor were installed in the base, and the pitch angle of the electric push rod of the undulating device was realized by controlling the stepping motor in the base. By controlling the DC motor in the base of the electric push rod to drive the gear to rotate, the free expansion and contraction of the electric push rod is realized. Through the combination of the lodging mechanism and the sensor, a more flexible observation angle can be provided for the robot’s ability to perform environment detection.

### 2.4. Firefighting Robot System Block Diagram

**Figure 7:**
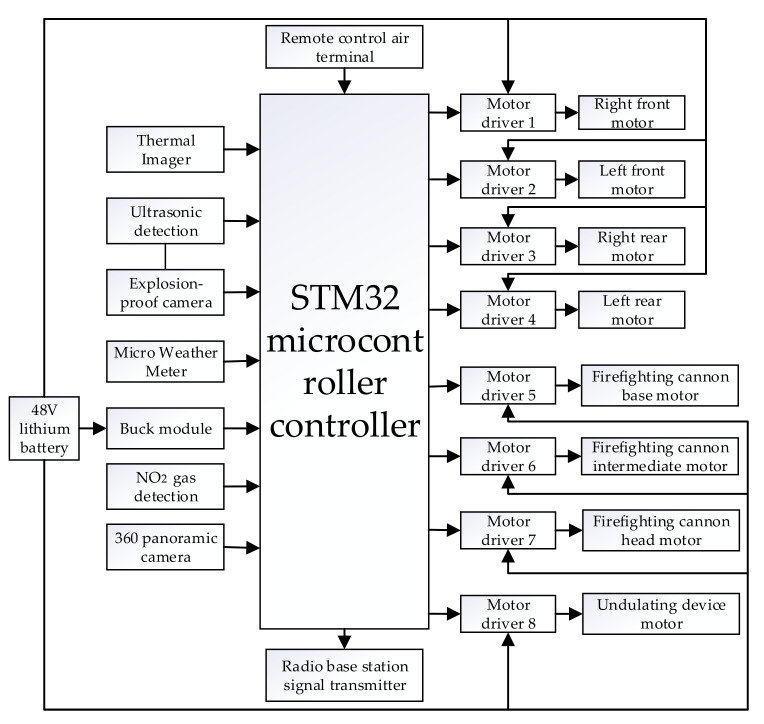
Robot system layout.

The overall control of the firefighting robot was programmed through the MDK program development environment and through the STM32F103 microcontroller as the central control unit. The Figure 7 below shows the general layout of the robot system.

### 2.5. Sensors Mounted on Firefighting Robots

**Figure 8:**
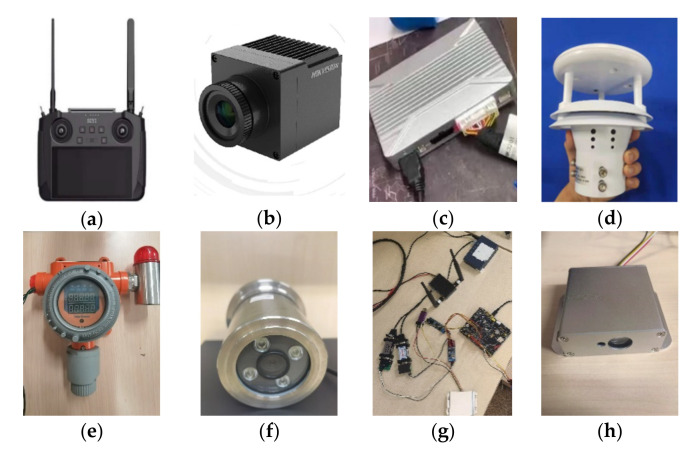
Sensors on the robot. (**a**) Remote control. (**b**) Thermal Imager. (**c**) Panoramic camera. (**d**) Micro weather meter. (**e**) Gas detection. (**f**) Explosion-proof camera. (**g**) Micro radio. (**h**) Laser rangefinder.

Due to the needs of the project, the firefighting robot was equipped with a 360 panoramic camera, NO_2_ gas detection device, thermal imager, high-definition explosion-proof camera, miniature weather instrument, and ultrasonic detection device, as shown in Figure 8. Additionally, the robot was equipped with a miniature radio base station. After the information is detected by the laser rangefinder and gas detector in the sensor, it is analyzed by the STM32 controller, and the controller outputs the analyzed data through the serial port of the transmitter of the radio base station. The detected information is sent to the receiver of the radio base station, which is convenient for the operator to detect remotely. The image information from the thermal imager and explosion-proof camera is directly transmitted remotely through the image transmission system of the radio base station to the receiving end. The 360 panoramic camera transmits images through the transmission system of the remote control, which is convenient for decision makers to operate the robot. The theoretical maximum transmission distance of the remote control system is 3.5 km. The miniature weather instrument can be used to detect the temperature, humidity, wind speed, air pressure, and other information about the current environment of the robot. The instrument came with a SIM card and can send the weather information to the remote end through the network.

## 3. Robot Kinematic Analysis

In the complex terrain environment, the non-tracked robots have the best comprehensive motion performance [17]. The crawler robots have many combinations and transformations they can make due to their crawler composite configuration, and they are divided into many types. This firefighting chassis consisted of four-track assemblies, with each track assembly consisting of a driving wheel, a regulating wheel, and a supporting wheel. Each driving wheel is connected to the car body through a bearing, which can realize the speed adjustment of the four tracks in different directions. The crawler robots are very similar to a four-wheel-drive robot [18,19,20]. Four-track assemblies can replace four driving wheels equivalently, and the steering of the body is all sliding steering. Specifically, the basic principles of four-track robot steering and four-wheel-drive robot steering are the same, and both are realized by controlling the relative speeds of the tracks (or wheels) on both sides. Therefore, the mathematical model of the four-track robot is established. It can be simplified to the mathematical model of the four-wheel-drive robot for analysis.

### 3.1. Coordinate System Establishment

Based on the above analysis, since the basic motion principle of the four-track robot steering is similar to that of the four-wheel-drive robot, the way of establishing the coordinate system is also similar. The coordinate system is also established with the center of mass as the origin. In the low-speed motion state, the four-wheel-drive robot realizes the steering motion through sliding friction, thus the influence of the robot’s mass distribution on the robot’s movement needs to be considered, and the four-track robot also takes the center of mass as the origin. To create a coordinate system, first, the motion model is simplified when: (1) the wheel of the robot does not spin when rolling; (2) the mass of the robot body is evenly distributed, and (3) the center of mass (COM) is located on the geometric longitudinal symmetry line of the robot, but not necessarily on the geometric lateral symmetry line.

**Figure 9:**
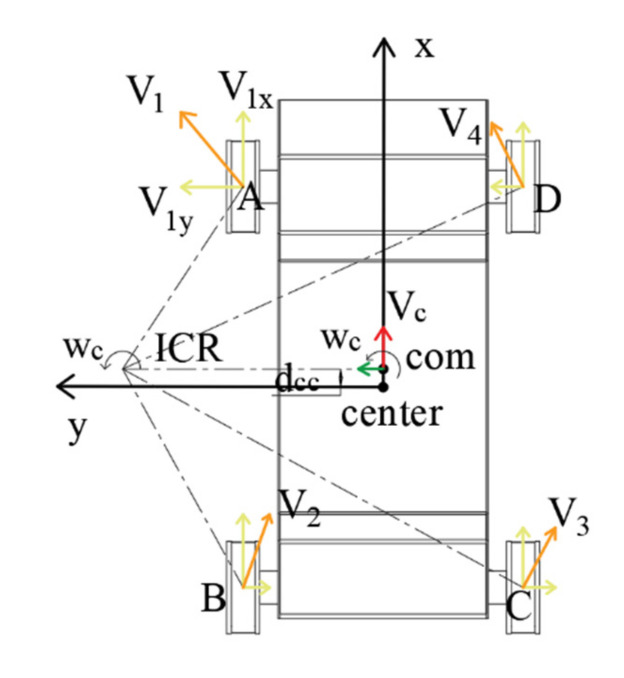
Body coordinate system diagram.

The crawler robot can be regarded as the rigid body, which is approximated and simplified as the ideal model in Figure 9. The ICR (instantaneous center of rotation) in Figure 9 indicates that the rigid body has only rotational motion around this point. As shown in the figure above, the coordinate system X-CENTER-Y is established with the geometric center of the robot as the origin, and the coordinate system X-COM-Y is established with the center of mass COM as the origin. The *X*-axes of the two coordinate systems coincide, the Y-axes are parallel, and the COM to the distance to CENTER is dcc. The forward movement direction of the robot is the positive direction of the *X*-axis (red arrow), the positive direction of the *Y*-axis (green arrow) is perpendicular to the left, and the Z-axis is perpendicular to the outside of the paper, which satisfies the right-hand rule.

### 3.2. Motion Model Law Analysis

Similar to the non-omnidirectional constraints of the two-wheel differential-drive robot, the four-track robot only describes its motion through the linear velocity and angular velocity [$v_{c}$ $\omega_{c}$]^T^, and the speed space of the two is also similar; therefore, the coordinate system is established. The definition of velocity space is consistent with the convention of velocity direction. The point COM has only the linear velocity along the *X*-axis, as well as the angular velocity, and the ICR is located on the Y-axis of the coordinate system X-COM-Y. It can be seen from Figure 9 that the combined velocity directions of the ideal contact points (A, B, C, and D) of the track and the ground are all perpendicular to the radial direction of the rotation radius, and can be decomposed into the longitudinal sub-velocities along the rolling direction of the track and lateral speed along the motor axis.

#### 3.2.1. Analysis of Longitudinal Velocity of Left Wheel

**Figure 10:**
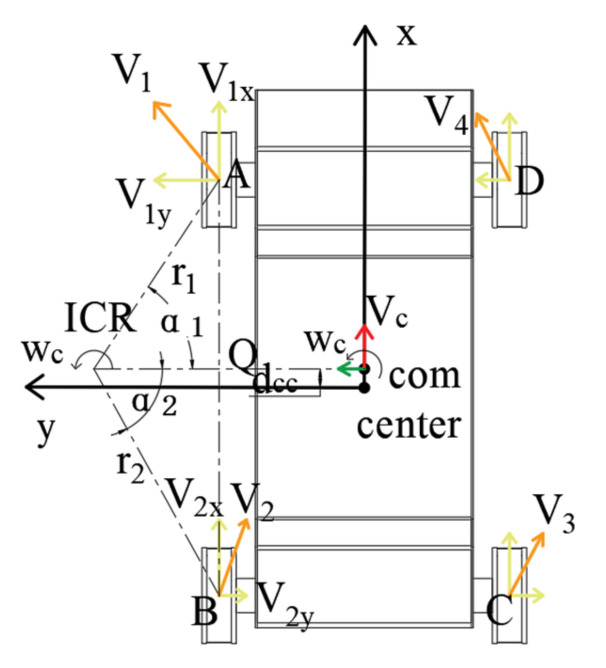
Left wheel analysis diagram.

As shown in Figure 10, the speed of the two left-track assemblies at the point of contact with the ground can be expressed as:

(1) $$\omega_{c}=\frac{v_{1}}{r_{1}}=\frac{v_{2}}{r_{2}}$$

Among them, $v_{1}$ and $v_{2}$ represent the linear speeds of points A and B, respectively, and $r_{1}$ and $r_{2}$ correspond to their turning radii. In RTΔA-Q-ICR and RTΔB-Q-ICR, the triangular transformation is used to convert Formula (1), which is expressed as:

(2) $$\frac{v_{1}}{\left\|Q-ICR\right\|/\cos\alpha_{1}}=\frac{v_{2}}{\left\|Q-ICR\right\|/\cos\alpha_{2}}$$

In Formula (2), $\alpha_{1}$ and $\alpha_{2}$ represent the included angles of A-ICR, B-ICR, and Q-ICR. After further simplification of Formula (2), it is expressed as:

(3) $$v_{1x}=v_{1}\cos\alpha_{1}=v_{2}\cos\alpha_{2}=v_{2x}$$

In Formula (3), $v_{1x}$ and $v_{2x}$ represent the sub-velocities of $v_{1}$ and $v_{2}$, respectively. Based on the above analysis, it can be seen that the longitudinal sub-velocities of points A and B are the same. Similarly, it can be analyzed that the longitudinal sub-velocities of points C and D are the same, but the longitudinal sub-velocities of the left and right crawler tracks are different.

#### 3.2.2. Analysis of Lateral Component Velocity of Front-Crawler Assembly

**Figure 11:**
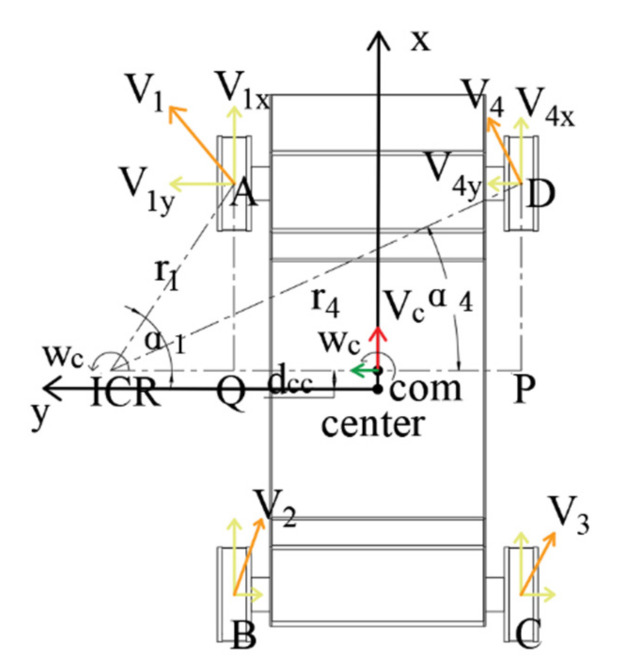
Front wheel analysis diagram.

Using the same idea of the analysis method of longitudinal velocity of the track assemblies on both sides, as shown in Figure 11, the velocity of the contact point between the two front wheels and the ground can be expressed as:

(4) $$\omega_{c}=\frac{v_{1}}{r_{2}}=\frac{v_{4}}{r_{4}}$$

where $v_{4}$ represents the linear velocity of point D, and $r_{4}$ corresponds to its turning radius. In RTΔA-Q-ICR and RTΔD-P-ICR, the triangular transformation is used to convert Formula (4), which is expressed as:

(5) $$\frac{v_{1}}{\left\|AQ\right\|/\sin\alpha_{1}}=\frac{v_{1}}{\left\|DP\right\|/\sin\alpha_{4}}$$

In the Formula (4), $\alpha_{1}$ and $\alpha_{4}$ represent the angles between A-ICR, D-ICR, and P-ICR. 

Because AQ and DP have the same length, Formula (5) is further simplified and expressed as:

(6) $$v_{1y}=v_{1}\sin\alpha_{1}=v_{4}\sin\alpha_{4}=v_{4y}$$

In Formula (6), $v_{1y}$ and $v_{4y}$ represent the lateral component velocities of $v_{1}$ and $v_{4}$ about the *Y*-axis, respectively. 

Based on the above analysis, it can be seen that the lateral component velocities of points A and D are the same. Similarly, it can be analyzed that the lateral component velocities of points B and C are the same, but the lateral component velocities of the front and rear wheels are different.

#### 3.2.3. Regular Summary

Through the analyses in the above Section 3.2.1 and Section 3.2.2, the regular equation is transformed into the following equation:

(7) $$\left\{\begin{array}{c} v_{l}=v_{1x}=v_{2x}\\ v_{r}=v_{3x}=v_{4x}\\ v_{f}=v_{1y}=v_{4y}\\ v_{b}=v_{2y}=v_{3y} \end{array}\right.$$

In Formula (7), $v_{l}$ and $v_{r}$ represent the longitudinal component speeds of the left and right wheels, respectively, and $v_{f}$ and $v_{b}$ represent the lateral component speeds of the front- and rear-track assemblies, respectively.

### 3.3. Kinematic Model Establishment

**Figure 12:**
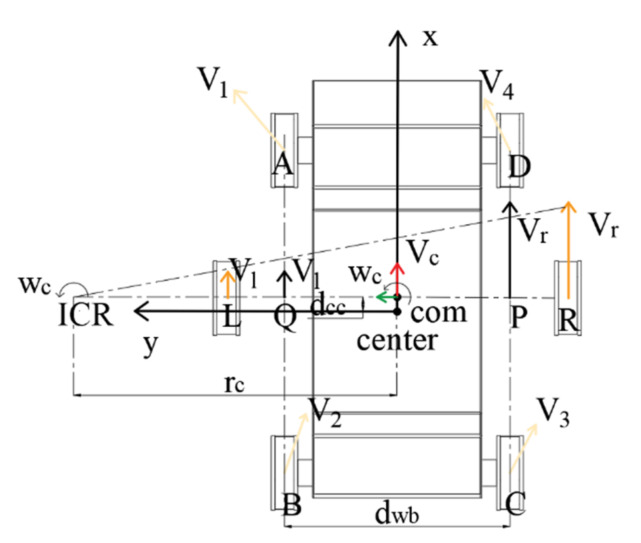
Simplified model diagram.

The movement of a four-track robot is equivalent to a four-wheel-drive mobile robot, thus the kinematic model can be simplified as the kinematic model of a two-wheel differential-drive robot. As shown in Figure 12, taking ICR-COM as the horizontal axis and CENTER-COM as the vertical axis, suppose the positions of the virtual left and right wheels are located at points L and R, respectively. The length of the virtual wheel spacing LR is not necessarily equal to the length of the real wheel spacing QP, and the virtual wheel spacing LR changes dynamically: if there is no rotational motion (no slip), it means that there is only $v_{l}$ and $v_{r}$, and if $v_{f}$ and $v_{b}$ are both 0, then LR = QP; if there is rotational motion, that is, $v_{f}$ and $v_{b}$ are not 0, then the actual situation has changed, which is to say that the angular velocity calculated according to $v_{l}$, $v_{r}$, and PQ is not the real angular velocity, the rotation sub-motion is different, and the slip (sliding friction) at different degrees has different effects on the actual angular velocity. Therefore, the virtual wheel spacing LR changes dynamically and is related to the sliding friction. Therefore, the contact between the ground and the tire with different friction coefficients has different effects on the actual rotational motion.

In the simplified two-wheel differential movement model, the linear velocity direction of the left and right virtual wheels of the robot is the same as the x-axis, and the linear velocity direction is perpendicular to the rotation radius, thus the ICR must be located on the line connecting points L and R. The specific position on the straight line LR is determined by the left and right driving wheel speeds [$v_{1}$ $v_{r}$].

Since v=ω×r, when ω is constant, v and r are proportional. Therefore, the speeds of points L, R, and the point COM can be expressed as:

(8) $$\omega_{c}=\frac{v_{c}}{r_{c}}=\frac{v_{r}}{r_{c}+d_{wb}/2}=\frac{v_{l}}{r_{c}-d_{wb}/2}$$

The velocity satisfies the similar triangle relationship: it can be seen from Formula (8) that since the four points of L-R-COM-ICR are collinear, the velocities of points L, R, and COM satisfy the similar triangle relationship in Figure 12; therefore, the geometric meaning of ω is the tangent value at vertex ICR. According to the deformation of Formula (8), the angular velocity ω can be expressed as:

(9) $$\omega =(v_{r}-v_{l})/d_{wb}$$

Angular velocity ω direction: when $v_{l}$ < $v_{r}$, ω > 0; otherwise, ω < 0.

Further, the relationship between the linear speed $v_{c}$ of the point COM and the left and right virtual wheel speeds [$v_{l}$ $v_{r}$] can be calculated through the deformation of Formula (8):

(10) $$v_{c}=(v_{r}+v_{l})/2$$

Further, the turning radius $r_{c}$ of point COM can be expressed as the following relationship:

(11) $$r_{c}=\frac{v_{c}}{\omega_{c}}=\frac{(v_{l}+v_{r})d_{LR}}{2(v_{r}-v_{l})}$$

In Formula (11), $d_{LR}$ represents the virtual wheel spacing.

Expressing the kinematic model with a virtual equivalent model, the simplified model of the four-wheel-drive mobile robot is expressed as:

(1) The simplified forward kinematic model calculates the speed of the geometric center of mass COM based on the speed of the virtual left and right driving wheels, which can be expressed as:

(12) $$\left[\begin{array}{c} v_{c}\\ \omega_{c} \end{array}\right]=\left[\begin{array}{c} \frac{v_{r}+v_{l}}{2}\\ \frac{v_{r}-v_{l}}{d_{LR}} \end{array}\right]=\left|\begin{array}{cc} 1/2 & 1/2\\ 1/d_{LR} & -1/d_{LR} \end{array}\right|\left[\begin{array}{c} v_{r}\\ v_{l} \end{array}\right]$$

(2) The simplified inverse kinematic model decomposes the speed of the left and right driving wheels based on the speed of the geometric center of mass COM, which can be expressed as:

(13) $$\left[\begin{array}{c} v_{r}\\ v_{l} \end{array}\right]=\left[\begin{array}{c} v_{c}+\frac{d_{LR}}{2}\omega_{c}\\ v_{c}-\frac{d_{LR}}{2}\omega_{c} \end{array}\right]\left|\begin{array}{cc} 1 & \frac{d_{LR}}{2}\\ 1 & -\frac{d_{LR}}{2} \end{array}\right|\left[\begin{array}{c} v_{c}\\ \omega_{c} \end{array}\right]$$

## 4. Robot Climbing Ability Analysis and Obstacle-Climbing Posture Analysis

When the robot performs tasks, it will encounter some complex terrains. These special terrains test whether the robot can successfully complete the task. Therefore, it is necessary to analyze the robot’s climbing ability and side parking on the slope. The conclusion shows that the robot has a good obstacle-surmounting posture when it drives over obstacles, which greatly improves the obstacle-surmounting ability.

### 4.1. Robot Climbing Ability Analysis

Climbing ability has always been a very important performance index for robots, and the climbing ability is usually measured by the maximum climbing angle [21]. The robot drive motor can drive normally only when it overcomes various resistances, such as when ignoring air resistance:

(14) $$F_{q}=F_{f}+F_{s}$$

In Formula (14), *F_f_* and *F_S_* can be solved in an iterative way:

(15) $$\left\{\begin{array}{c} F_{f}=fmg\cos\alpha \\ F_{s}=mg\sin\alpha \end{array}\right.$$

From Formulas (14) and (15),

(16) $$F_{q}=F_{f}+F_{s}=mg(f\cos\alpha +f\sin\alpha )$$

In Formula (16):

$F_{q}$—motor driving force;

$F_{f}$—frictional resistance;

$F_{s}$—uphill resistance;

f—friction coefficient;

m—the total mass of the robot;

g—gravitational acceleration, take 9.8 m/s^2^;

α—slope angle.

Generally speaking, the climbing ability of the robot is restricted by three aspects: the driving force of the motor, the adhesion to the ground, and the anti-overturning ability. The following analyzes the climbing performance of the robot from three aspects: the driving force of the motor, the adhesion to the ground, and the static longitudinal stability of the robot.

#### 4.1.1. Analysis of Climbing Ability Affected by Motor Driving Force

**Figure 13:**
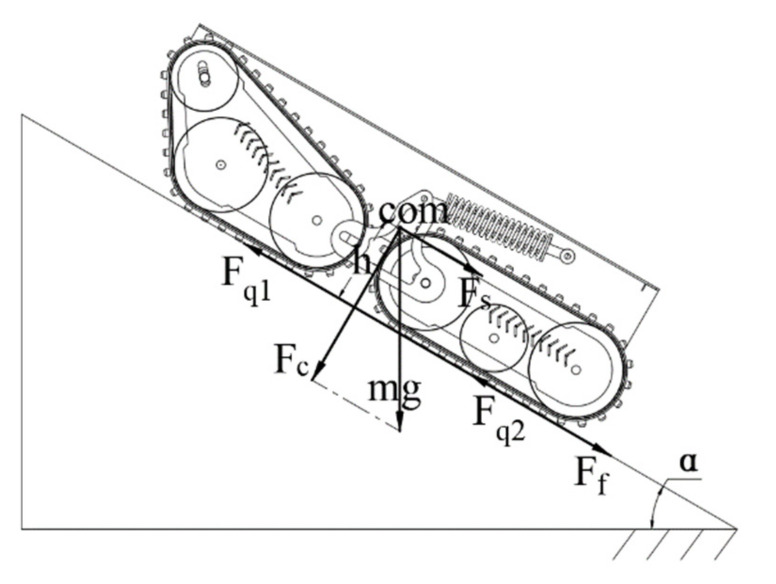
Force diagram of the robot on the slope.

The mechanical analysis of the robot climbing ability affected by motor driving force is shown in Figure 13. The maximum driving force of the motor is:

(17) $$F_{\max}=\frac{3600\;p\eta }{V}$$

In Formula (17):

*p*—motor power at the maximum torque reached by the motor (KW);

*η*—mechanical transmission efficiency, 0.94~0.98, take 0.96;

*V*—speed when the motor reaches the maximum torque (km/h).

Substitute Formula (16) into Formula (17) to obtain:

(18) $$f\cos\alpha +f\sin\alpha \leq \frac{3600\;p\eta }{mgV}$$

Set φ=arctanf, according to the formula of the auxiliary angle, and the maximum climbing angle affected by the driving force of the motor can be obtained:

(19) $$\alpha \leq \arcsin\frac{3600\;p\eta }{mgV\sqrt{1+f^{2}}}-\arctan f$$

#### 4.1.2. Analysis of the Influence of Ground Adhesion on Robot Climbing Ability

**Figure 14:**
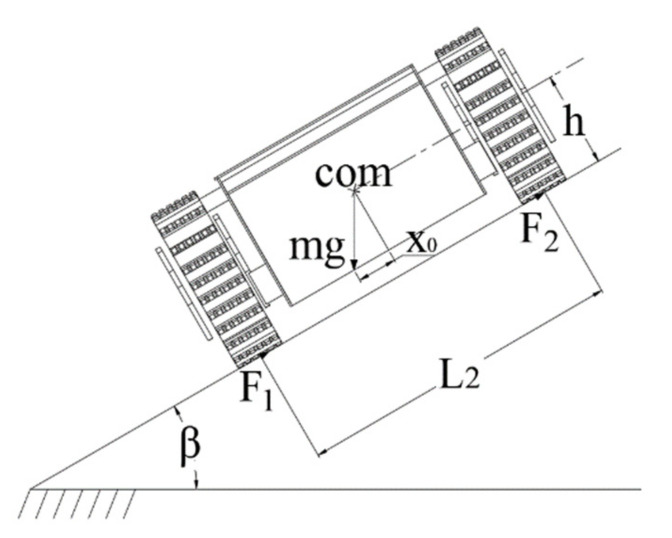
The lateral force diagram of the robot on the slope.

The robot is driven back and forth, as shown in Figure 14, and the driving force must meet:

(20) $$F_{q}=F_{q1}+F_{q2}=kmg\cos\alpha$$

In Formula (20):

$F_{q}$—total driving force;

$F_{q1}$—front wheel drive;

$F_{q2}$—rear wheel drive;

*K*—ground attachment coefficient, take 1.2.

From Formula (20), it can be known that when the driving force and the quality of the robot are constant, the maximum climbing angle under the influence of the adhesion force is:

(21) $$\alpha_{\max}=\arccos\frac{F_{q}}{k_{\max}mg}$$

If the driving force is greater than the static friction force, the excess driving force is released by slipping, which causes the robot to slip when climbing and results in transmission failure and damage to the track, especially on hard roads. At the same time, mechanical energy is wasted. If the driving force is less than the friction force, the robot cannot move forward because the static friction cannot be overcome, and the motor is easily damaged. Therefore, in order to protect the motor and enable effective transmission, the motor needs to provide sufficient driving force. Therefore, when selecting the track, factors such as working road conditions, track material, track tooth shape, and driving speed should be comprehensively considered to enlarge the adhesion coefficient as much as possible. The robot is driven by a drive motor with a power of 1000 W and a maximum speed of 3000 r/min. After calculation, the maximum climbing angle is 36°.

#### 4.1.3. Lateral Slope Stability under Gravity

In a narrow space and complex terrain environment, the firefighting robot travels laterally on ramps. When the slope angle is greater than the tipping angle threshold, the vehicle overturns or slides laterally, and the chassis of the firefighting robot parks on the slope [22]. The maximum slope angle is called the transverse slope slip angle $\beta_{max}$, and the force diagram of the fire robot chassis on the transverse slope is shown in Figure 14.

When the robot rolls over, the reaction force on the right side is 0, and the critical value *β* of the rollover angle satisfies:

(22) $$mgh\sin\beta =\frac{L_{2}}{2}mg\cos\beta$$

(23) $$\beta =\arctan\frac{L_{2}}{2h}$$

In Formulas (21) and (22):

$L_{2}$—track gauge, mm;

$x_{0}$—lateral offset of the resultant force of the ground normal reaction force, mm.

The maximum slope angle at which the chassis of the firefighting robot does not slip when parking on a cross slope is $\beta_{max}$.

The force balance equation is:

(24) $$mg\sin\beta =F_{1}+F_{2}$$

(25) $$mg\cos\beta =k(F_{1}+F_{2})$$

The conditions for the chassis of the firefighting robot not to slip are:

(26) mgsinβ<kmgcosβ

(27) $$\beta_{\max}<\arctan k$$

In Formula (26):

*k*—ground attachment coefficient, take 1.2;

$F_{1}$—lateral adhesion of the left-track wheel, N;

$F_{2}$—lateral adhesion of the right-track wheel, N.

### 4.2. Analysis of Obstacle-Crossing Attitude of Robot Chassis

A good attitude planning can make the robot’s motor torque smaller, the overall center of mass move smoothly, and the robot pass through the obstacles smoothly. The process of the fire robot climbing the steps requires the center of mass to continuously improve, and finally let the center of mass cross the outer edge of the step [23]. The posture of the robot climbing the steps of different heights is similar. It is only possible that the rotation angle of the box is different, but in general, it can be divided into three stages, which is similar to the behavior when going up the slope because the slope can be seen as many superimposed combinations of the steps; however, they are somewhat different, and consist of the stage of the “Head up phase” and the stage of the “bending phase”.

#### 4.2.1. Four-Drive, Four-Track Joint Obstacle-Crossing Pose Analysis

**Figure 15:**
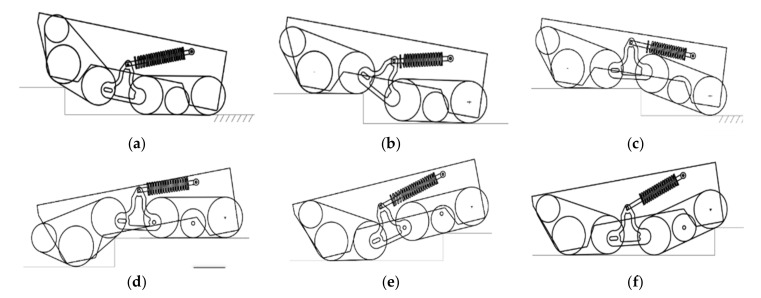
The analysis diagram of the robot’s obstacle-crossing posture. (**a**) The front track climbs the steps. (**b**) The front track goes over the steps. (**c**) The rear track goes over the steps. (**d**) The front track begins to climb over the steps. (**e**) The front track goes over the steps. (**f**) The rear track goes over the steps.

The pose diagram of the robot chassis climbing the steps is shown in Figure 15. Stages (a)–(c) are the “Head up phase”, in which the robot track makes contact with the steps to climb over. In Figure 15a, the front-crawler assembly is in contact with the step to climb over, the rear-crawler assembly is still moving in a plane, the car body is rotated clockwise by a certain angle, and the height ratio of the center of mass is higher than the height of the step. The front-track assembly rotates clockwise to a certain angle, and the connecting piece pushes the compression spring for shock absorption. In Figure 15b, the front-crawler assembly moves in a plane after climbing over the upper steps, the rear crawler is slightly raised by a certain angle, the spring is also compressed, and the center of mass of the car body continues to increase. In Figure 15c, the front-track assembly moves in a plane, the rear-track assembly rotates in contact with the step connecting piece, the spring is pulled to relax and further stretch the spring, and the center of mass of the car body is too high. Eventually, the entire robot climbs over the steps.

Figure 15d,e are the “bending phase”, in which the robot climbs down the stairs. In Figure 15d, the front-track assembly climbs over the steps first, but does not leave the step completely. The rear-track assembly is still moving in the plane, the car body rotates counterclockwise, and the center of mass begins to drop. At this time, the connecting piece rotates clockwise to cause compression and spring damping. In Figure 15e, the front-track assembly completely drives down the step, and then moves in a plane. The rear-track assembly part moves away from the step, the center of mass of the car body further drops, the connecting piece rotates counterclockwise, and the spring is released and stretched from the compressed state of the previous step. In Figure 15e, the car body continues to move forward, the front-crawler assembly moves in a plane, most of the rear-crawler assembly moves away from the steps, the rear-crawler assembly rotates counterclockwise around the rear bearing, the connecting piece rotates clockwise, the spring begins to be squeezed from the stretched and relaxed state of the previous step, and finally, the entire car body climbs down the steps. By analyzing the posture state of the robot over the steps, it can be seen that the walking mechanism connects the front and rear tracks through the movable connecting piece joints, so that the robot not only ensures the superior obstacle-surmounting ability of the track structure when overcoming obstacles, but also provides a certain stable effect for the top of the car body; the mechanism makes the robot more suitable for performing firefighting tasks in complex environments.

#### 4.2.2. Comparison of Four-Track Joint Structure and Traditional Fixed Track

**Figure 16:**
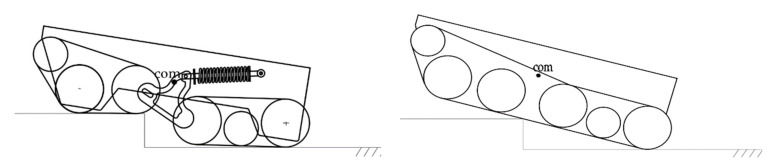
Obstacle-crossing posture comparison chart.

The right side of Figure 16 shows the posture diagram of the robot chassis using the traditional fixed-crawler structure to climb over the steps. The left side shows the pose diagram of the robot chassis using the four-drive, four-track articulated structure chassis to climb over the steps. It can be seen from the comparison of obstacle-crossing postures of different track structures in Figure 16 how the robot that adopts a traditional fixed-crawler structure has a center of gravity that is high when overcoming obstacles, and the contact area between the crawler and the ground is very small; thus, the robot has poor stability and a large tilt angle of the robot body. After adopting the four-track articulated structure, the position of the robot’s center of gravity becomes lower, the contact area between the track and the ground is larger when the robot goes over obstacles, and the stability is improved. Additionally, the tilt angle of the top of the robot is reduced, which is more conducive to the installation of other firefighting mechanisms on the top of the robot chassis. By comparing the poses of the two mechanisms when crossing obstacles, it can be seen that the designed four-track, four-drive articulated crawler structure has superior obstacle-crossing performance and stability.

## 5. ADAMS Simulation Analysis of Robot Mobile Chassis

### 5.1. Model Building

#### 5.1.1. Mobile Chassis ADAMS Model Construction

**Figure 17:**
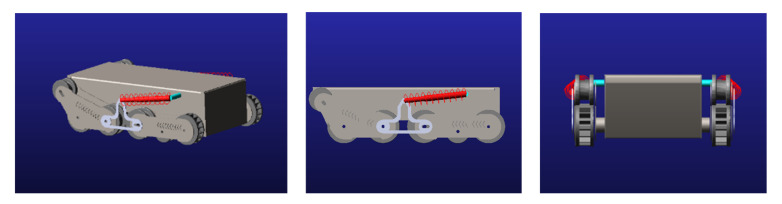
ADAMS model of the robot chassis.

The car body model was drawn and simplified in Solidworks. Since there are too many constraints to directly import the Solidworks model into ADAMS, it is difficult to perform motion simulation directly. Therefore, the various parts of the car body were simplified in Solidworks and saved in the Parasolid format, and the model was directly imported into ADAMS through the Parasolid format. Since the crawler is a flexible body, when adding the crawler, the contact constraints between the crawler and the ground are very complex, and the number of related constraints added is very large; thus, the simulation is prone to errors. Therefore, it is easy to complete the simulation by removing the track during the simulation, and the simulation results are basically the same as the simulation with the track added. In the simplified model, the main body is the car body, and four-track assemblies are added to the body. Each track assembly consists of a driving wheel, an adjusting wheel, a supporting wheel, and a side plate. In the model, a rotating pair is added between the driving wheel and the vehicle body for connection. Since the driving wheel, support wheel, and adjustment wheel of the crawler assembly are driven by the rubber track in the real entity, in the model, the driving wheel, adjustment wheel, and support wheel are connected by a coupling pair to achieve the effect of pulley transmission. Spring constraints are added between the front- and rear-track assemblies and the car body for analysis. Through the above steps, the simulated car body model was built in ADAMS, as shown in Figure 17.

#### 5.1.2. ADAMS Simulation Terrain Construction

**Figure 18:**
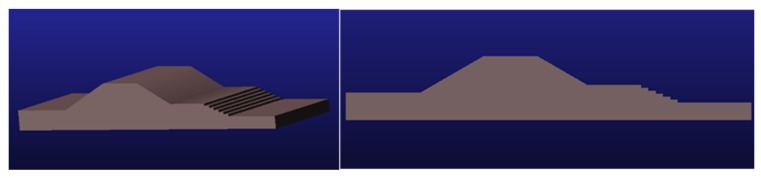
ADAMS simulation topographic map.

Considering the application of the car body in a real scene, the car body may pass through steps, slopes, and other sites; thus, steps and slopes are added to the terrain for simulation. Under the national standard, the step width of indoor and outdoor steps of public buildings should not be less than 0.3 m, and the step height should not be greater than 0.15 m, and should not be less than 0.1 m. Therefore, the height of the steps in the terrain was selected as 0.12 m, and the width of the steps was selected as 0.3 m to build a step terrain simulation. The slope angle was set to 30° so that the car body goes through the climbing stage and the downhill stage for analysis, and the terrain was built as shown in Figure 18.

### 5.2. Static Analysis

**Figure 19:**
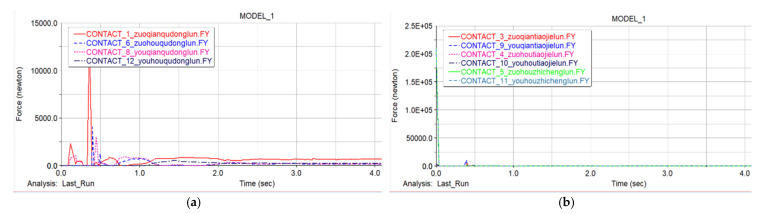
Track wheels and ground contact force. (**a**) The contact force of the driving wheels. (**b**) The contact force of other track wheels.

When the body rests on the ground, the body is subjected to gravity to generate a contact force with the ground, and the contact force generated by the contact between the body and the ground is analyzed by ADAMS. It can be seen from the model that only the support wheels in the two front assemblies of the machine are not in contact with flat ground, and the remaining track wheels generate contact force between the track and the ground. Figure 19a shows the contact force between the four front and rear driving wheels and the ground. The simulation results in the first second of the vibration are due to the force generated, but the contact force tends to become stable after one second. It can be seen that the four front and rear wheels have a contact force between the driving wheel and the ground of about 200–300 N. Figure 19b shows the force situation of the remaining pulleys in contact with the ground. Through analysis, it can be seen that in the entire robot chassis, the four driving wheels mainly provide support for the car body, and the overall stability of the robot is good.

### 5.3. Kinematic Simulation

**Figure 20:**
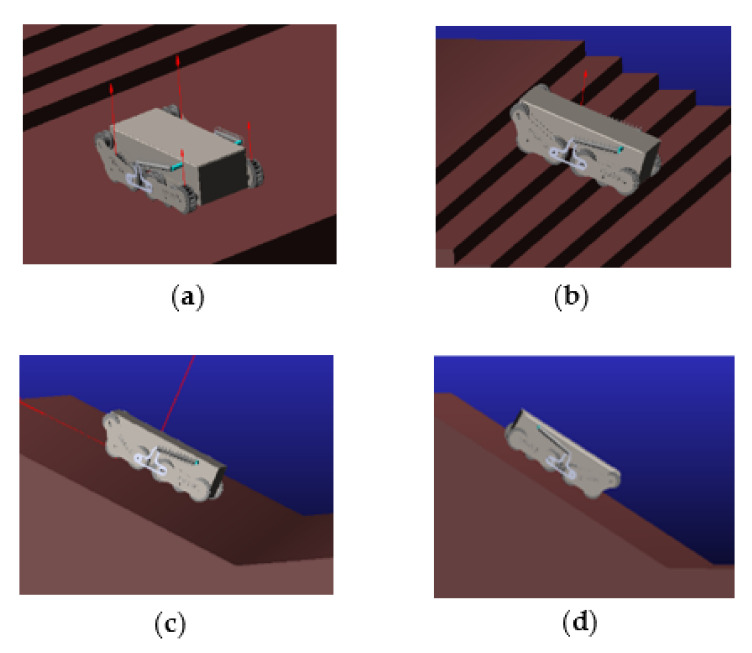
Obstacle-crossing map of different terrains. (**a**) The chassis moves forward on flat ground. (**b**) The chassis climbs on the steps. (**c**) The chassis runs up the ramp. (**d**) The chassis runs down the ramp.

The firefighting robot is mainly responsible for detecting the environment and carrying out firefighting operations in a narrow space. Therefore, it is necessary to have certain requirements for the robot’s ability to overcome obstacles. The ADAMS simulation simulates the movement of the car body over terrain such as steps and conducts the climbing and descending slope analysis. The images of the car body at different terrain stages are shown in Figure 20. In the figure, the motion pictures in different terrains built by the robot chassis in ADAMS were intercepted. The motion of the robot simulation chassis on the flat ground, shown in Figure 20a, shows that the robot chassis moves smoothly. In Figure 20b, the robot chassis begins to climb the steps. In Figure 20c, the robot chassis climbs the 30-degree slope. In Figure 20d, after the robot drives to the top of the slope, it runs through the flat ground and starts downhill along the 30° slope. In the end, the robot chassis completes the moving simulation of flat ground and steps that go uphill and downhill.

Table 1 is the robot motion timetable. According to the kinematic simulation figures, the motion state of the car body at different times was counted. At 9.1 s, the car body began to touch the steps, completely climbed the steps at 15 s, started climbing at 16s, completed the climbing at 20 s, started downhill at 22 s, and completed the downhill climb at 25 s.

### 5.4. ADAMS Simulation Results Analysis

#### 5.4.1. Motion Analysis of Shock Spring

**Figure 21:**
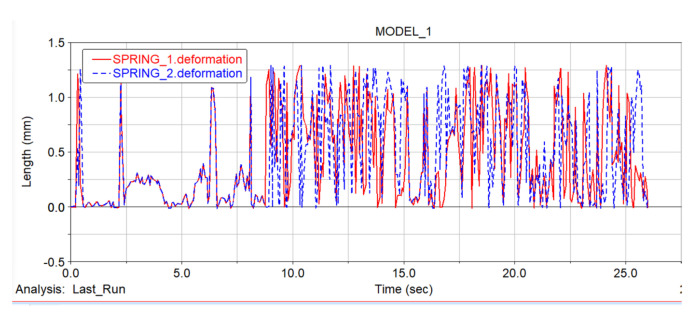
Left and right spring deformation diagram.

Figure 21 shows the comparison of the deformation of the shock-absorbing springs on the left and right sides in the robot dynamic simulation experiment. The red curve represents the deformation curve of the left spring, and the blue curve is the deformation curve of the right spring. Combining the motion time analysis, it can be seen that the time with the largest deformation difference is the “step climbing stage” in the simulation process. When the simulation started at 0 s, the car body was in contact with the ground under the action of gravity, which is equivalent to the car body falling and being in contact with the ground. After the spring was deformed, it tended to be stable. At 9 s, the car body started to climb the steps. Through the attitude analysis of the vehicle body, it can be seen that it basically conformed to the deformation state of the spring in the attitude analysis. During the step-climbing stage, the spring was continuously pulled up and squeezed, and the deformation degree of the spring was mainly affected by the spring damping. From 15 s to 16 s and 20 s to 22 s, the car body was in the “Flat ground movement stage”. At this time, the movement state of the car body changed, and the spring deformation was affected by the movement state of the front section. There were certain fluctuations, but the overall orientation was in a stable state. At 16 s to 20 s and 22 s to 25 s, the car body was climbing and descending, and the spring was deformed. During these two periods, the spring deformation fluctuation was weaker than that of the “step climbing stage”. It can be seen from Figure 21 that there was a slight delay between the deformation of the left and right springs. According to the analysis, a certain delay occurred when the left and right crawler were climbing, as one crawler climbed to a certain height first, but it did not affect the overall overturning state of the car body.

**Figure 22:**
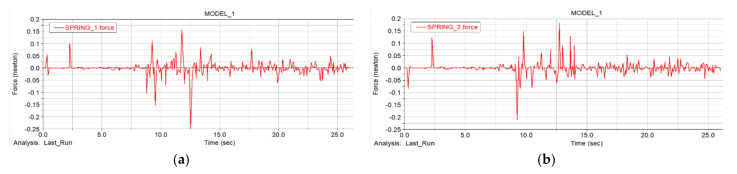
Force diagram of left and right springs. (**a**) The force diagram of the left spring. (**b**) The force diagram of the right spring.

Figure 22 shows the force analysis diagram of the left and right shock absorber springs. Combined with the analysis of the spring deformation diagram, it can be seen that the spring stress hardly changed much in the “Flat ground movement stage”. At the beginning, the car body was in contact with the ground due to gravity and started to move forward under the action of the driving force, and the spring tended to stabilize after a certain fluctuation. The stage where the spring stress fluctuated violently is the “step climbing stage”. At this time, the maximum force of the spring in tension and compression deformation under different motion states of the front and rear tracks was 0.2 N.

Overall, the shock absorber spring realized the shock absorption effect between the front- and rear-track assemblies and the car body when the car body went over obstacles, and realized the posture change of the car body during the climbing stage. From the comparison of the springs on the left and right sides, it can be seen that the tension and compression of the springs on both sides were not uniform when climbing the steps. The reason for this state is that due to the different friction between the track and the terrain, when the front-tracks on the left and right sides climbed over the steps, one track climbed to a certain height first, and the opposite side spring was squeezed. However, it did not affect the robot’s obstacle crossing. 

By analyzing the deformation and force of the shock absorber spring, it can be seen that when the robot is overtaking obstacles, the spring plays a certain role in the deformation between the front- and rear-track assemblies, making the deformation of the track joints more flexible and stable, and improving the chassis obstacle-crossing stability.

#### 5.4.2. Car Body Speed Curve Analysis

**Figure 23:**
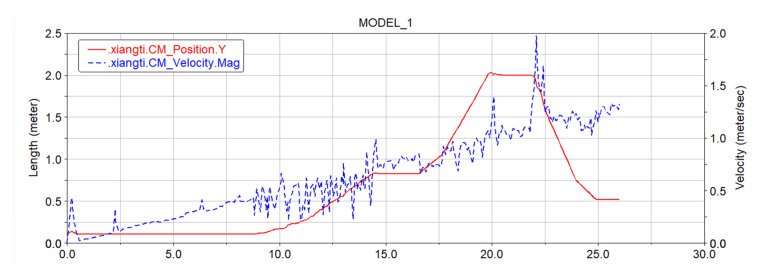
Spatial position and velocity curve of robot centroid.

In Figure 23, the blue curve is the velocity curve of the center of mass of the car body, and the red curve is the curve of the center of mass of the car body changing with time in the *Y*-axis direction. By selecting the height curve of the center of mass of the car body, it is more convenient to observe the motion state of the car body at different stages, including the curve and the terrain. The curve is similar to the topographic map for easy observation. From the analysis of the graph, it can be seen that at the beginning of the simulation, the vehicle speed fluctuated rapidly under the action of the driving force when the vehicle body touched the ground. After that, the vehicle body was completely in stable contact with the ground and began to be in the “Flat ground movement stage”, in which the vehicle speed increased steadily under the action of the driving force. When the “step climbing phase” began, the vehicle body was affected by obstacles, and the vehicle speed fluctuated violently, but the overall speed was still in the increasing phase. In the “Ramp climbing stage” and “Lower slope stage”, the vehicle speed fluctuated to a certain extent under the action of gravity and friction on the slope, but the vehicle speed was still increasing. It can be seen from the figure that two special stage points were at 20 s and 22 s. At this time, the speed of the vehicle changed drastically. The reason for the analysis is that the vehicle body speed did not decelerate when it reached the peak and started to descend the slope, and the vehicle body was partially vacated. If the vehicle body decelerates when it reaches the top of the slope and goes downhill, there would be violent fluctuations.

**Figure 24:**
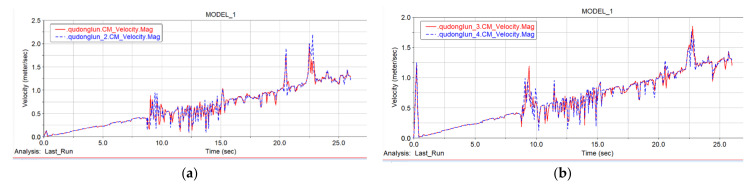
Front and rear driving wheel speed. (**a**) Front driving wheel speed. (**b**) Rear driving wheel speed.

Figure 24a shows the speed curve of the front driving wheels, where the red curve is the speed curve of the left driving wheel, and the blue curve is the speed curve of the right driving wheel. Since the vehicle body is of a symmetrical structure, when performing speed analysis in combination with the terrain, the front- and rear-track assemblies are compared and analyzed separately. Figure 24b shows the speed comparison and analysis diagram of the left and right driving wheels of the front track. From the figure it can be seen that when moving on the ground at the initial stage, the speeds of the left and right driving wheels remained the same. In the “step climbing phase” at 9:00, the front right wheel speed reached a peak before the speed of the left front wheel. It can be seen that when the first step was being climbed, the right front crawler climbed the steps before the left front crawler, thus the speed of the left front wheel was delayed to a certain extent compared with the speed of the right front wheel. The main reason for this phenomenon is the influence of the friction factor between the ground and the track, but it did not affect the climbing of the car body. Judging from the speed curves of the first two driving wheels in the image, the speed of the driving wheels fluctuated to a certain extent due to the terrain, but the speed change curves of the driving wheels on the front side of the car body were basically the same. The left and right driving wheels had a slight lag phenomenon due to the friction between them and the terrain, but the overall speed was constantly increasing.

**Figure 25:**
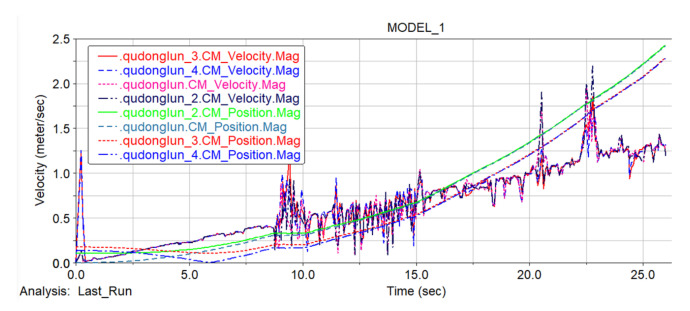
The speed and space position curve of the center of mass of the driving wheel.

In Figure 24b, the red curve is the speed curve of the left rear driving wheel, and the blue curve is the speed curve of the right rear driving wheel. The speed change of the rear-side driving wheel of the vehicle body was similar to the speed change of the front-side driving wheel. The fluctuating curve at the bottom in Figure 25 is the speed curve of the four driving wheels, and the smoother curve at the top is the spatial position curve of the center of mass of the four driving wheels changing with time. It can be seen from the figure that despite the changes of the vehicle body speed and position, the speed of the driving wheels was basically the same. In the whole simulation, the maximum speed reached 2.3 m/s, but this speed was the speed when the robot continued to accelerate and leapt over the top of the slope. After the car body touched the ground again, the speed stabilized at 1.4 m/s and the simulation ended. 

It can be seen from the analysis of the velocity curve of the center of mass of the vehicle that the robot chassis has excellent mobility and obstacle-surmounting ability.

## 6. Prototype Verification

In order to verify the function and obstacle-crossing ability of the designed firefighting robot, the designed firefighting robot was prototyped. After the prototype was installed, the parameters are shown in the following Table 2. The theoretical driving mileage of the prototype was calculated by the prototype’s motor and battery parameters, and the movement of the prototype under different terrain conditions was tested, including three-step obstacle crossing, slope crawling, and different ground movement tests.

### 6.1. Robot Chassis Theoretical Movement Distance

The robot’s moving speed is calculated according to the rated speed and the radius of the driving wheel:

(28) $$V=\pi \times d\times n_{R}$$

(29) $$n_{R}=\frac{n}{I}$$

(30) $$V=\pi \times d\times \frac{n}{I}$$

In Formulas (27)–(29):

*V*—robot speed, m/s;

*d*—driving wheel diameter, m;

*n*—the rated speed of the drive motor, r/min;

*I*—reducer reduction ratio;

*n_R_*—reducer speed; m/s.

To calculate the theoretical discharge time and theoretical rating distance of the battery according to the rated current:

(31) $$T=\frac{Ah}{I_{N}}$$

(32) S=V×T

In Formulas (30) and (31):

*T*—battery theory continuous discharge timed, h;

*Ah*—battery capacity;

*I_N_*—rated current, a;

*S*—robot theoretical movement distance, m.

After calculation, the theoretical movement distance of the robot chassis was 8573 m; in the actual situation, the robot’s moving distance was less than the secondary theoretical movement distance. With the research and development of today’s new batteries, the capacity of batteries is increasing, and battery volume is decreasing. The robot’s moving distance can be extended by selecting a more excellent battery, or having the robot use a backup battery as a replacement, which can increase the working time of the robot.

### 6.2. Prototype Different Terrain Test

**Figure 26:**
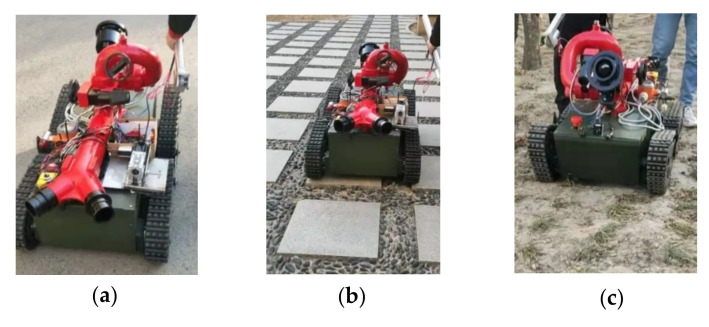
Robot moving test on different ground. (**a**) Asphalt road. (**b**) Floor tiles and gravel pavement. (**c**) Dirt pavement.

The prototype vehicle was driven out of the room and walking and obstacle-crossing tests were conducted. In the walking test, the driving state of the prototype vehicle under different road conditions was tested. In the obstacle-crossing test, the ability of the prototype vehicle to climb over obstacles was tested. To carry out the walking test, first, the driving of the prototype vehicle was tested under road surface conditions. The prototype car ran smoothly on the road surface under the control of the remote control, performing the functions of forward, backward, steering, etc., and there was no slippage; then, traveling on an uneven road surface with road and floor tiles was tested. On these two kinds of roads, the prototype car ran normally, but had a certain degree of shaking; however, the steering, and forward and reverse functions were realized. The test images are shown in Figure 26.

**Figure 27:**
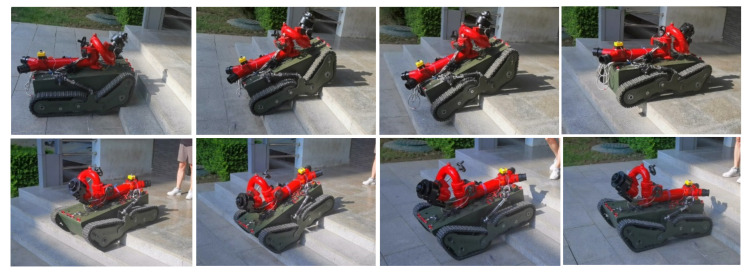
Robot climbing steps.

Obstacle-surmounting ability tests in the step terrain were conducted, by controlling the prototype car to drive out of the laboratory with the remote control. There was a three-step terrain at the entrance to the building, with each step being 0.155 m high and 0.33 m wide. The angle of the three steps was about 35°. The obstacle-crossing process is shown in Figure 27. The robot was able to climb up and down the steps smoothly.

**Figure 28:**
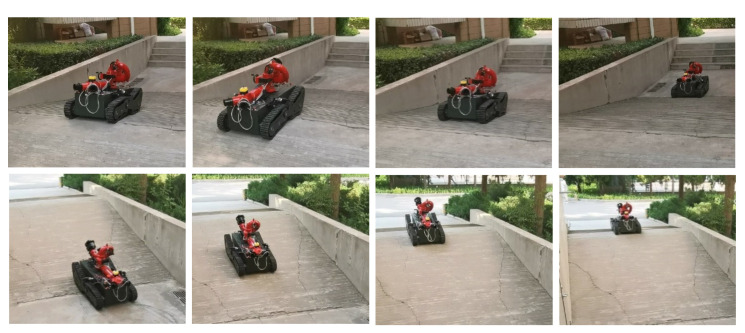
Robot ramp movement.

The robot moved on a sloped terrain for testing; however, due to the limited test site, it was difficult to find a suitable slope on the campus. Finally, the slope surface of the following Figure 28 was found and used for the slope test, where the slope height was about 1 m, and the slope length was 3.5 m. The obstacle angle was about 16°, and the robot ran stably at this angle slope.

**Figure 29:**
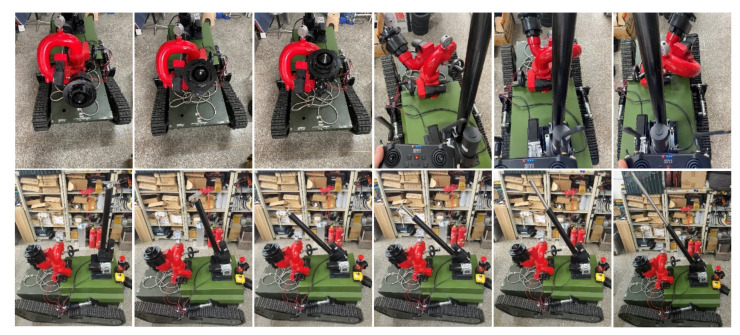
Robotic firefighting cannon and undulating device test.

Finally, the fire monitor and undulating device test were carried out, and the experimental results are shown in Figure 29.

Through the remote control handle, the firefighting cannon was controlled up and down, left and right, and to open and close. At the same time, the telescopic and multi-angle movement of the undulating device could be controlled. The firefighting cannon and the undulating device test passed. The prototype vehicle completed the task requirements and realized various functional requirements.

## 7. Conclusions

The new, confined firefighting robot designed in this paper adopts a four-track and four-drive articulated crawler structure for walking, which has a strong ability to overcome obstacles. The front and rear tracks are connected by movable connecting pieces and equipped with shock-absorbing springs, so that the robot not only has superior obstacle-surmounting ability, but also provides a certain degree of stability when overcoming obstacles. Through the installed sensors, both the body chassis and the fire monitor can be remotely operated with a panoramic stereoscopic view, which is convenient for the firefighting cannon to spray at different angles and replace the operator to operate tasks in a dangerous environment. The vehicle body integrates a variety of sensor information, and can transmit the surrounding environment information and sensor data of the vehicle to the remote control and decision-making receiver through the image transmission system and the transparent transmission system to help with remote operation and decision making. In the future, the deep learning-based technique [24,25,26] will be examined to improve the performance of this articulated tracked firefighting robot.

## Figures and Tables

**Table 1:** Robot motion timetable.

Starting Time	End Time	Movement Stage
0	1	At the beginning of the simulation, the car body touches the ground and moves forward under the action of the driving force under the influence of gravity.
1	9	Flat ground movement stage.
9	15	Step climbing stage.
15	16	Flat ground movement stage.
16	20	Ramp climbing stage.
20	22	Flat ground movement stage.
22	25	Lower slope stage.

**Table 2:** Some parameters of the prototype.

Information	Parameter
Height	900–1360 mm (undulating mechanism retracts)
Width	750 mm
Length	1200 mm
Quality	Approximately 250 kg
Battery specifications	72v50ah
Chassis drive motor rating Parameters	Rated power 1000 w; rated current 26.4 A Rated speed 3000 rpm
Reducer reduction ratio	1:25
Driving wheel diameter	0.2 m
Theoretical remote control distance	10–15 km

## Data Availability

The data are available upon request.
